# Nonsurgical deep uterine transfer of vitrified, *in vivo*-derived, porcine embryos is as effective as the default surgical approach

**DOI:** 10.1038/srep10587

**Published:** 2015-06-01

**Authors:** Emilio A. Martinez, Cristina A Martinez, Alicia Nohalez, Jonatan Sanchez-Osorio, Juan M. Vazquez, Jordi Roca, Inmaculada Parrilla, Maria A. Gil, Cristina Cuello

**Affiliations:** 1 Department of Animal Medicine and Surgery, University of Murcia, Spain

## Abstract

Surgical procedures are prevalent in porcine embryo transfer (ET) programs, where the use of vitrified embryos is *quasi* non-existent. This study compared the effectiveness of surgical vs nonsurgical deep uterine (NsDU) ET using vitrified, *in vivo*-derived embryos (morulae and blastocysts) on the reproductive performance and welfare of the recipients. The recipient sows (n = 122) were randomly assigned to one of the following groups: surgical ET with 30 vitrified-warmed embryos (S-30 group, control); NsDU-ET with 30 vitrified-warmed embryos (NsDU-30 group) and NsDU-ET with 40 vitrified-warmed embryos (NsDU-40 group). Regardless of embryo stage, the NsDU-ET with 40 embryos presented similar rates of farrowing (72.7%) and litter size (9.9 ± 2.1 piglets) as the customary surgical procedure (75.0% and 9.6 ± 2.7 piglets). Numbers of ET-embryos appeared relevant, since the NsDU-ET with 30 embryos resulted in a decrease (P < 0.05) in farrowing rates (38.9%) and litter sizes (5.7 ± 2.4 piglets). In conclusion, we demonstrate for the first time that farrowing rate and litter size following a NsDU-ET procedure increase in function of a larger number of transferred vitrified embryos, with fertility equalizing that obtained with the invasive surgical approach. The results open new possibilities for the widespread use of non-invasive ET in pigs.

Embryo transfer (ET) technology has many potential applications in pig production, including the movement and on-farm introduction of new genetic material (i.e. embryos) with reduced transportation costs, no effect on animal welfare during transport and minimal risk of disease transmission. In spite of these benefits, the practical use of ET in pigs, unlike other species, is currently extremely limited or *quasi* non-existent owing to for the need of performing surgery for embryo deposition and the difficult embryo cryopreservation. In the past decade, however, new methodologies have been devised to overcome these hurdles; nonsurgical ET and embryo vitrification.

Nonsurgical ET in non-sedated/anesthetized female pigs was considered a non-viable technique for many years because of the complex anatomy of their genital tract that made impossible its passage with anything but liquids. We developed a unique procedure for the nonsurgical transfer of embryos deep into the uterine horn (NsDU) of non-sedated, basically unrestrained gilts or sows. During the first attempt of NsDU-ET with fresh embryos, an acceptable reproductive performance (71.4% farrowing rate and 6.9 piglets born) was achieved^1^. With procedural improvements, these results were greatly enhanced^2^,^3^ and excellent farrowing rates (90%) and litter sizes (9.0 pigs) were reported even following NsDU-ET of fresh morulae, cultured for 24 h *in vitro*^4^. Although a 24 h-culture period allow for international shipment of embryos, cryopreservation and the ability to store them for extended periods of time is preferable. Development of a repeatable technique to cryopreserve pig embryos of morula and blastocyst stages would provide other transcendental applications for the pig industry, including indefinite embryo storage, increased selection pressure in select herds, rescue of premium genetics from diseased herds and international export/import of potential breeding stocks^5^. Vitrification is the only currently suitable method for long-term storage of porcine morulae or blastocysts, reaching today high post-warming *in vitro* survival even without embryo pre-treatments (e.g., delipidation, cytoskeletal stabilization, and centrifugation) (for review see Ref. 6). Transfer of these vitrified embryos, albeit surgical, had reached promising farrowing rates and litter (for review see Ref. 6), but such development is hampered by the need of large numbers of embryos available at one particular time, the use of an invasive technique for ET and all the high costs and welfare issues involved.

The use of NsDU-ET instead of surgical ET, coupled with embryo vitrification for storage of available embryos for ET are thus essential if swine ET is to be widespread as technique for genetic gain. We tested their combined use in previous experiments, where we obtained acceptable farrowing rates (40–50%) and litter size (5–10 piglets)^7^,^8^. However, the number of recipients used was low thus calling for further research to confirm the effectiveness of NsDU transfer of vitrified embryos. In addition, as with the development of any new technology, evaluation of the specific factors that affect the success rate of NsDU-ET is needed. One of these items is to establish which number of vitrified embryos to be transferred is optimal for the technique, translated into optimal farrowing rates and litter size in recipients. To the best of our knowledge, no studies have addressed this issue using either surgical or NsDU transfers. The only available study used recipients that were slaughtered after 30 days of gestation, and where no differences were detected in rates of pregnancy or embryo survival with 20 surgically transferred vitrified blastocysts compared with 30^9^. Such attempts needed confirmation, since independent researchers found that surgical transfer with 25-35 vitrified embryos^10^,^11^ resulted in larger litter size than when only 20 vitrified embryos were transferred^12^,^13^.

The objectives of the present study were (i) to compare the effectiveness of surgical and NsDU transfers of vitrified porcine embryos and (ii) to determine whether the number of vitrified embryos transferred affected the reproductive performance and welfare of the recipients after NsDU-ET.

## Methods

### Chemicals

The chemicals used in this study were purchased from Sigma-Aldrich Quimica SA (Madrid, Spain) unless indicated otherwise.

### Animals

This work was conducted in field conditions at a commercial pig farm located in Southeastern Spain (Murcia, Spain). Weaned crossbred sows (Landrace×Large-White) from the same genetic line (1-6 parity) were used as embryo donors and recipients. The sows were kept individually in crates in a mechanically ventilated confinement facility. The semen donors were sexually mature boars (2-3 years of age) housed in climate- controlled individual pens (20–25 °C) at a commercial insemination station in Murcia (Spain). Animals had *ad libitum* access to water and were fed commercial diets according to their nutritional requirements.

All the experimental procedures used in this study were performed in accordance with Directive 2010/63/EU EEC for animal experiments and were reviewed and approved by the Ethical Committee for Experimentation with Animals of the University of Murcia, Spain (research code: 638/2012).

### Detection of estrus and insemination

Only sows with a weaning-to-estrus interval of 4 to 5 days were selected as donors or recipients. The detection of estrus was performed once a day beginning two days after weaning by allowing snout-to-snout contact of females with vasectomized mature boars and by application of back pressure by experienced personnel. Animals that showed a standing estrous reflex were considered to be in estrus and were used in the experiment.

The donors were post-cervically inseminated at 6 and 24 h after the onset of estrus. The insemination doses (1.5 × 10^9^ spermatozoa in 45 mL) were prepared from sperm-rich fractions of the ejaculates extended in Beltsville thawing solution extender^14^ and were stored for a maximum of 72 h at 18 °C.

### Embryo recovery and evaluation

The collection of embryos was performed in a specifically designed surgical room located on the farm. The donors were subjected to a mid-ventral laparotomy on Day 6 of the estrous cycle (Day 0: onset of estrus). The donors were sedated with azaperone (2 mg/kg body weight, intramuscular). General anesthesia was induced using sodium thiopental (7 mg/kg body weight, intravenous) and was maintained with isoflurane (3.5-5%). After exposure of the genital tract, the corpora lutea on the ovaries were counted. The embryos were collected by flushing the tip of each uterine horn with 30 mL of Tyrode’s lactate (TL)-HEPES-polyvinyl alcohol (PVA)^15^ with some modifications^4^. The recovered embryos were evaluated under a stereomicroscope at a magnification of 60× to grade the developmental stage and quality. One-cell eggs and poorly developed embryos were classified as unfertilized oocytes and degenerated embryos, respectively. The remaining embryos with the appropriate morphology according to the criteria determined by the International Embryo Transfer Society^16^ were considered viable. Vitrification was only performed on compacted morulae and unhatched blastocysts with morphology graded as excellent or good.

The ovulatory response of the donors was determined by counting the number of corpora lutea on both ovaries. Recovery rate was defined as the ratio of the number of embryos and oocytes and degenerated embryos recovered to the number of corpora lutea present. Fertilization rate was defined as the ratio of the number of viable embryos at collection to the total number of embryos and oocytes and degenerated embryos collected.

### Vitrification and warming

The collected embryos were washed four times in TL-HEPES-PVA, placed in Eppendorf tubes containing 1.5 mL of the same medium and subsequently transported in an incubator at 39 °C in our laboratory at the University of Murcia. The embryos were washed 6 times in TL-HEPES-PVA at 39 °C before vitrification. Vitrification was performed within 5 h after collection. Groups of five to seven embryos were vitrified with the method described by Berthelot *et al.*^12^ and modified by Cuello *et al.*^17^. The embryos were handled in media at 39 °C. Vitrification and warming were accomplished using the chemically defined media described by Sanchez-Osorio *et al.*^18^ as basic medium (TCM199-HEPES supplemented with 0.1% PVA: TCM-PVA). For vitrification, the embryos were washed twice in TCM-PVA and sequentially equilibrated in the first vitrification medium (TCM-PVA + 7.5% DMSO + 7.5% ethylene glycol) for 3 min and in the second vitrification medium (TCM-PVA + 16% DMSO + 16% ethylene glycol + 0.4 M sucrose) for 1 min. In the final step, the embryos were placed in a 1-μL drop and loaded into the narrow end of a superfine open pulled straw (SOPS; Minitüb, Tiefenbach, Germany) by capillary action. Subsequently, the straws containing the embryos were plunged horizontally into liquid nitrogen (LN_2_).

After storage in LN_2_, the straws were removed and warmed by the one-step warming method^19^,^20^. Briefly, the straws containing the embryos were vertically submerged in the first well of a four-well, multi-dish plate (Nunc A/C, Roskilde, Denmark) containing 800 μL of TCM-PVA supplemented with 0.13 M sucrose and were equilibrated for 5 min.

### *In vitro* embryo culture and assessment of *in vitro* embryo development

To evaluate the *in vitro* development of the embryos, some vitrified-warmed embryos were cultured for 72 h in a four-well, multi-dish plate containing 500 μL of embryo culture medium composed of NCSU-23^21^ supplemented with 0.4% bovine serum albumin and 10% fetal calf serum at 39 °C in humidified air with 5% CO_2_. The embryos were evaluated morphologically every 24 h during culture with a stereomicroscope to determine developmental progression. Morulae that progressed to the blastocyst stage during *in vitro* culture and those blastocysts that reformed their blastocoelic cavities after warming and displayed a normal or thinning zona pellucida, with an excellent or good appearance, were considered viable. *In vitro* survival rate was defined as the ratio of viable embryos after culture to the total number of cultured embryos. *In vitro* hatching rate was defined as the ratio of hatching and hatched embryos at the end of the culture to the total number of cultured embryos.

### Embryo transfer

Immediately after warming, embryos were loaded into Tomcat catheters for transfer into the recipients. The Tomcat catheter was loaded with air bubbles that separated the 30-μL drop of medium that contained the embryo from two drops of TCM-PVA before and after the embryo.

All ET were conducted in asynchronous (-24 hours to embryo collection) recipients. One hour before the transfer, each recipient received a single intramuscular injection of a long-acting amoxicillin suspension (Clamoxyl LA; Pfizer, Madrid, Spain) at a dose of 15 mg/kg.

The NsDU-ETs were performed with a previously described method^1^,^2^,^3^. Briefly, the recipients were housed in gestation crates in field conditions. The perineal area of the recipients was thoroughly cleaned, and the vulva was then washed and decontaminated with chlorhexidine. Nonsurgical ET catheters (Deep Blue ET catheter; Minitüb, Tiefenbach, Germany) were used for the transfers. When the ET catheter was completely inserted into one uterine horn, the Tomcat catheter containing the embryos was connected to the ET catheter, and the contents were introduced into the ET catheter with a 1 mL syringe. An additional volume of 300 μL of TCM-PVA was used to force the embryos out of the ET catheter into the uterus. During the transfer, the operator made a prediction of whether the insertion of the ET catheter was adequate (catheter well inserted deep into a uterine horn) or inadequate (bad location of the catheter into the uterine horn, curled up into the uterus). After transfer, correct positioning of the catheter was assumed if no bends or kinks in the ET catheter were present after its removal. The behavior of the females and the difficulties encountered during the insertion of the ET catheter were recorded.

The surgical transfers were conducted using the same procedure described previously for embryo collection. The embryos were transferred to the tip of a uterine horn (5 to 6 cm from the uterotubal junction) with a Tomcat catheter inserted through the uterine wall, which was previously punctured with a blunt Adson forcep.

Post transfer, all recipients were evaluated daily for behavioural changes, including signs of estrus beginning at 12 days post ET. Pregnancy was diagnosed by ultrasonography on Days 20 to 22 post-transfer. All pregnant sows were allowed to carry litters to term, and all variables (farrowing rate, litter size, numbers of piglets born alive, sex of the offspring and birth weight) were recorded. Piglet production efficiency was calculated as the ratio of the number of live-born piglets to the number of embryos transferred to all recipients.

### Experimental design

A total of 256 donors were selected based on their reproductive history (fertility: 86.4%; litter size: 11.0 ± 2.7 piglets; parity number: 5.9 ± 1.0; and length of lactation: 21.6 ± 2.4 days). The embryos were collected in 26 trials over a 1.5 year period, and each trial consisted of 7-12 donors. The donors of each individual trial were inseminated with sperm from the same boar. The embryos (compacted morulae and unhatched blastocysts) were vitrified and stored in LN_2_ for at least three months. The recipients (n = 122) were randomly assigned to one of the following groups: surgical ETs with 30 vitrified-warmed embryos (S-30 group); NsDU-ETs with 30 vitrified-warmed embryos (NsDU-30 group); and NsDU-ETs with 40 vitrified-warmed embryos (NsDU-40 group). The recipients were selected based on their reproductive history and body condition. There were no differences in the reproductive history of recipients assigned to each group [fertility (range: 84.4% to 87.1%), litter size (range: 10.8 ± 2.2 to 11.2 ± 2.6 piglets), parity number (range: 2.4 ± 1.7 to 2.6 ± 1.6) and length of lactation (range: 21.3 ± 2.6 to 21.7 ± 2.5 days)]. The vitrified-warmed embryos from each donor in each trial were equally and randomly allocated to each of the recipient groups. The ETs were performed by the same operator in a total of 16 trials, and each trial included 6 to 9 transfers. In each trial, several warmed embryos were randomly selected for *in vitro* culture immediately after warming to evaluate *in vitro* development, whereas the rest were transferred to the recipients.

### Statistics

The data were analyzed with the IBM SPSS 19 Statistics software package (SPSS, Chicago, IL, USA). The percentage data were compared using Fisher’s exact test. Continuous variables were evaluated using the Kolmogorov-Smirnov test to assess the assumption of normality, and groups were compared with analysis of variance. Post hoc analysis was performed using Bonferroni’s test. The CV (SD/mean) was used as a measure of variability of the ovulatory response. Differences were considered significant when P < 0.05. The results are expressed as percentages and means ± SD.

## Results

Of the 256 donors, 93.7% (n = 240) had embryos on Day 6 post-insemination. The mean ovulation rate obtained in the fertilized sows was 23.3 ± 3.8 corpora lutea (range: 12 to 31 corpora lutea, CV = 16.3%). The recovery rate was 93.0%, and the mean number of viable embryos, and oocytes and degenerated embryos ascended to 19.4 ± 3.4 and 2.3 ± 1.8, respectively (89.4% fertilization rate). The proportion of vitrificable embryos (compacted morulae and unhatched blastocysts) in relation to the number of viable embryos was 94.3%. Non-vitrifiable viable embryos were exclusively related to the presence of uncompacted morulae and hatched blastocysts. A total of 4,390 vitrificable embryos were collected, of which 2,375 (54.1%) and 2,015 (45.9.2%) were classified as compacted morulae and unhatched blastocysts, respectively. Of these embryos, 313 were used for the evaluation of *in vitro* development after warming, and the remaining embryos were used for ET.

The *in vitro* viability of vitrified-warmed morulae and blastocysts after *in vitro* culture is shown in Table 1. Although a large percentage of vitrified-warmed embryos survived the vitrification procedure, there was a significant effect (P < 0.001) of the stage of embryo development on post-warming viability, with blastocysts having a higher *in vitro* survival and hatching rates than morulae.

The predictive diagnosis on the location of the NsDU-ET catheter into the uterus was considered as correct in 84.1% of the transfers. Thirteen out of 82 NsDU-ETs were removed from the study because of incorrect insertion of the NsDU-ET catheter. The difficulties encountered during the insertion of the ET catheter were considered as none or minor in 98.5% of the transfers. The behavior of the females during the procedure was classified as good (no reaction) in all cases. No vaginal discharges were observed after the procedure in any of the recipients. The reproductive performance of recipients after transfer is shown in Table 2. The highest pregnancy and farrowing rates and litter size (total piglets born and piglets born alive) were achieved in the S-30 and NsDU-40 groups, with no differences between these groups. However, a notable decrease in these variables occurred (P < 0.05) among recipients in the NsDU-30 group.

A total of 10 sows (14.3%, 17.6% and 7.4% for the S-30, NsDU-30 and NsDU-40 groups, respectively) lost their pregnancy between Days 25 and 35 with an irregular return to estrus (Days 27 to 34). All sows pregnant on Day 35 farrowed, except one sow from the NsDU-40 group that aborted on Day 54 of pregnancy. Piglet production efficiency was higher (P < 0.004) in the S-30 group compared with the NsDU-ET groups. The lowest (P < 0.001) piglet production efficiency was obtained in the NsDU-30 group. No differences in piglet birth weight or sex ratio per litter were observed among groups.

A total of 56, 48 and 5 ETs were performed with only morulae, only blastocysts or a blend group of morulae and blastocysts, respectively. The reproductive variables of recipients that solely received morulae or blastocysts in each experimental group are shown in Table 3. Within each group, embryonic stage transferred did not have any influence on the evaluated variables, exception made of the piglet production efficiency, which was higher (P < 0.03) for blastocysts in the NsDU-30 group.

## Discussion

This study is the first of its kind to demonstrate the effect that number of transferred vitrified-warmed embryos has on the reproductive performance of recipients following a NsDU-ET procedure, which is currently the only available method for the nonsurgical transfer of embryos deep into the uterine horn in pigs. Additionally, for the first time, we compared the effectiveness of surgical vs nonsurgical ETs using vitrified-warmed embryos. The relevance of the studies becomes higher when considering the trials were run under commercial conditions of swine production with a relatively high number of recipients.

To the best of our knowledge, only one report has assessed the most appropriate number of vitrified embryos needed to be transferred into a recipient^9^; an experimental study that evaluated pregnancy rates and number of fetuses 35 days after surgical transfer to a reduced number of recipients, thus differing notably from the present study. Those authors found no significant differences in pregnancy rates or in the mean number of viable fetuses on Day 30 of pregnancy irrespective of the number of vitrified embryos transferred (20 versus 30 embryos). They concluded that 20 vitrified embryos might be the optimal number of embryos for transfer to each recipient and that little benefit was gained from the transfer of more embryos. Surprisingly, such optimum numbers of embryos are even below those physiologically registered in the swine genital tract, following ovulation rates of 20-25 oocytes, usual in this species. It was speculated that the transfer of more that 20 embryos per recipient may be not justified due to the available uterine space for fetal development^9^. However, this speculation is unconvincing because the uterine space may be a factor in fetal death when large numbers of fetuses are present after Day 30^22^. This does not seem to be the case after transfer of vitrified embryos where the number of viable fetuses at Day 30 of pregnancy ranged 7 to 9^9^,^23^,^24^.

In contrast to the findings of Berthelot *et al.*^9^, the present results clearly demonstrate that litter size, as well as pregnancy and farrowing rates increased with a higher number (30 to 40) of vitrified-warmed embryos transferred by NsDU-ET. The protocols of these studies differed largely, as per method of vitrification (OPS vs. SOPS), the breed of the recipients (Meishan vs. Landrace × Large-White), the age of the recipients (gilts vs. sows) and the ET procedure (surgical vs. nonsurgical), all of which may have be behind the discrepancies registered. In any case, two previous findings support our results. Firstly, studies using surgical ETs with fresh embryos have shown pregnancy rates 20 to 30 points higher and mean numbers of normal viable embryos two- to three fold greater on Day 25 of gestation in recipients that received 24 embryos compared to those that only received 12^25^,^26^. Secondly, litter size was 1.5- to 3-fold larger after surgical or nonsurgical transfer of 27-35 vitrified embryos^8^,^10^,^11^ than after transfer of only 20 vitrified embryos^7^,^12^,^13^. Moreover, in the present study, piglet production efficiency in the S-30 group was 23.0%, which was comparable to previous reports that used surgical transfers with 25-30 vitrified embryos per recipient (range: 17.2% to 20.7%)^11^,^24^,^27^ yet higher than in studies with only 20 vitrified-warmed embryos (range: 9.5% to 13.0%)^12^,^13^. Additionally, in the present study, piglet production efficiency was notably increased in the NsDU-40 group (17.3%) compared with the NsDU-30 group (7.1%). Collectively, the data indicate that increasing the number of embryos transferred increases the number of viable fetuses at Day 30 of pregnancy, and subsequently, the number of piglets born alive.

In the present study, 30 vitrified-warmed embryos were adequate for surgical transfers but not for NsDU-ETs. However, in the NsDU-ET group with 40 vitrified embryos the reproductive performance of recipients was similar to that in the S-30 group. These results indicate that the number of vitrified-warmed embryos should be higher for nonsurgical ET than for surgical ET. This finding was not unexpected if we account for the situation with fresh embryos. Effectively, although there are no comparative studies, successful nonsurgical ET requires 24-30 fresh embryos per transfer^1^,^28^, almost double the number of fresh embryos recommended for surgical transfers^29^. A plausible explanation for the difference could be the location of embryo deposition, which is associated with the ET procedure used. In surgical ET, the embryos are deposited into the tip of the uterine horn^26^, whereas in NsDU-ET, the embryos are placed in the middle or anterior quarter of the uterine horn^1^,^6^,^7^,^30^. Under normal physiological conditions, the morulae and blastocysts remain near the tip of the uterine horn until Days 6 or 7 of the cycle and then progress toward the uterine body^31^. Therefore, it is reasonable to consider that the transfer of these embryos to the tip of the uterine horn might be advantageous compared to when embryos are placed in the more caudal portions of the uterus. Consistent with this hypothesis, results from surgical ETs indicate that the middle of the uterine horn is a more appropriate place than the caudal quarter of the uterine horn or the uterine body^32^. It is possible that the uterine environment in these regions is less favorable for embryos during these stages of development, resulting in impaired embryo survival or, alternatively, that some embryos are lost from the uterine cavity by ad-cervical myometrial contractions. In any case, the transfer of a higher number of embryos, as in the NsDU-40 group, had obviously overcome the negative effects listed above. More research is required to corroborate such hypotheses.

In the present study, vitrified embryos had high *in vitro* survival and hatching rates that were similar to those previously reported^8^,^18^,^20^,^27^,^33^,^34^,^35^. Consistent with previous data^36^, we obtained a significant effect of the stage of embryo development on embryo viability after warming, with morulae exhibiting lower *in vitro* rates of development than blastocysts. This result was neither unexpected, as developmental staging is one of the primary factors known to affect the tolerance of porcine embryos to vitrification^10^,^20^,^36^. Interestingly, we found that embryo stage (morulae and blastocysts) did not significantly affect farrowing rates or litter size of the recipients within each experimental group, despite the differences obtained in *in vitro* survival between vitrified morulae (75.0%) and blastocysts (90.7%). With these *in vitro* survival rates, the number of potential viable embryos transferred was 30 morulae and 36 blastocysts for the NsDU-40 group, which yielded an excellent farrowing rate (72.7%) and litter size (9.9 ± 2.1 piglets). These data are comparable to those recently reported by our laboratory using NsDU-ETs with 30 fresh viable morulae or blastocysts^2^,^3^,^4^. Therefore, we could hypothesize that morulae and blastocysts that survive vitrification have a similar ability to develop *in vivo* compared with fresh embryos at these developmental stages. However, more than 14%, 17% and 7% of recipients that were pregnant on Day 25 in the S-30, NsDU-30 and NsDU-40 groups, respectively, lost their pregnancy with an evident irregular return to estrus. Although the information available is very limited, the rates of pregnancy loss for vitrified embryos are typically between 10% and 20%^7^,^8^,^11^,^12^,^13^ compared with <2.5% for fresh embryos^1^,^2^,^3^,^4^. Albeit direct comparison among these studies must be applied carefully owing to their inherent experimental differences (donor and recipient genotypes, vitrification procedure, recipient parity, and number of recipients used, among others), the increase in pregnancy loss following transfers with vitrified embryos could be caused by a modification of gene and protein expression patterns in the placenta after implantation, a reduction in early fetal growth or an alteration of placental development, as it has been shown in rabbits^37^,^38^,^39^. However, other possibilities should also be considered. It is possible that the use of vitrified embryos resulted in pregnancies with a small number of surviving embryos or of embryos having lower mitotic rates than normal within the uterus, both of which might be sufficient for the maternal recognition of pregnancy to be expressed but not for the maintenance of pregnancy to term. Although the principal signal for recognition of pregnancy in pigs is estrogen secretion by embryos on Days 11 to 12 of pregnancy^40^, a second peak of embryo estrogen secretion occurs between Days 15 and 25 to 30^41^. An inadequate number of embryos at this later interval could result in a too low second peak of estrogen and, consequently, in pregnancy loss. More research is therefore necessary to determine the development of those vitrified embryos that implant successfully but which are unable to continue gestation to term.

We hereby used a unique technique for deep uterine nonsurgical embryo transfer in pigs, developed several years ago in our laboratory^1^. In 15% of the recipients, the catheter was incorrectly inserted into the uterine horn, as determined by the presence of kinks in the ET catheter after removal; thus leading to the exclusion of these sows from the study. These sows later returned to estrus on Days 20 to 22 of the cycle, indicating that the embryos were lost during the transfer, but that the ET-procedure, no matter how unsuccessful the embryo placement could have been, did not negatively impaired the reproductive physiology of the sows, returning to estrus at the expected, physiological interval. The overall correct prediction of the location of the catheter in the uterus was 84% (69 correct predictions out of 82 NsDU-ETs performed). These data are comparable to those achieved in our previous studies, where approximately 90% of the transfers were correctly predicted during the insertion of the ET catheter^2^,^3^. Therefore, from a practical perspective, the waste of embryos caused by incorrect insertion of the catheter in NsDU-ET trials should be considered. Most importantly, and consistent with our previous studies^2^,^3^,^4^, the insertion of the ET catheter was safe and well tolerated by the recipients and did not influence on the incidence of vaginal discharge post-transfer. The non-surgical procedure is definitively a safe, non-invasive and welfare-preserving reproductive technology for pig assisted reproduction.

In conclusion, our study demonstrated that, regardless of the stage of embryos (compacted morulae or unhatched blastocysts), NsDU-ETs with 40 vitrified embryos resulted in a notable increase in farrowing rates and litter size than when 30 embryos were transferred, reaching, non-surgically, the levels of performance obtained with the default technique, the invasive, surgical approach. Such good reproductive performance of recipients following NsDU-ETs with vitrified embryos represents an important advancement for the national and international trade of porcine embryos and opens news possibilities for the widespread use of ET in the pig industry.

## Additional Information

**How to cite this article**: Martinez, E. A. *et al.* Nonsurgical deep uterine transfer of vitrified, *in vivo*-derived, porcine embryos is as effective as the default surgical approach. *Sci. Rep.* **5**, 10587; doi: 10.1038/srep10587 (2015).

## Figures and Tables

**Table 1:** *In vitro* embryo survival and hatching rates of vitrified-warmed embryos after 48 h of culture.

**Embryo stage**	**Trials(N)**	**Warmed embryos (N)**	**Survival rate (mean** **±** **SD)**	**Hatching rate (mean** **±** **SD)**
Morulae	16	170	75.0 ± 5.0^a^	51.1 ± 8.1^a^
Blastocysts	16	143	90.7 ± 3.4^b^	73.4 ± 3.4^b^

^a,b^Different letters in the same column indicate differences (P < 0.001).

**Table 2:** Reproductive variables of recipients after surgical and nonsurgical deep intra-uterine embryo transfer of vitrified-warmed porcine embryos.

	Embryo transfer procedure*		
	**S-30**	**NsDU-30**	**NsDU-40**
No. of recipients	40	36	33
No. of parity	2.4 ± 1.7	2.6 ± 1.8	2.4 ± 1.5
Pregnancy rate (25 d), N (%)	35 (87.5)^a^	17 (47.2)^b^	27 (81.8)^a^
Pregnancy rate (35 d), N (%)	30 (75.0)^a^	14 (38.9)^b^	25 (77.8)^a^
Pregnancy length (d) (mean±SD)	115.0 ± 1.1	115.2 ± 2.1	115.4 ± 1.5
Farrowing rate, N (%)	30 (75.0)^a^	14 (38.9)^b^	24 (72.7)^a^
Total born (mean ± SD)	9.6 ± 2.7^a^	5.7 ± 2.4^b^	9.9 ± 2.1^a^
born alive (mean ± SD)	9.2 ± 2.5^a^	5.5 ± 2.4^b^	9.5 ± 2.2^a^
Piglet birth weight (Kg) (mean ± SD)	1.5 ± 0.3	1.6 ± 0.4	1.4 ± 0.2
Piglet production efficiency# (%)	23.0^a^	7.1^b^	17.3^c^

^*^S-30: Surgical transfers with 30 vitrified-warmed embryos; NsDU-30: Nonsurgical transfers with 30 vitrified-warmed embryos; NsDU-40: Non-surgical transfers with 40 vitrified-warmed embryos.

^#^Piglet production efficiency was calculated as the ratio of the number of live-born piglets to the number of embryos transferred to all recipients.

^a,b,c^Different letters in the same row indicate differences (P < 0.004).

**Table 3:** Effect of embryonic stage on the reproductive variables of recipients after surgical and non-surgical deep uterine transfer of vitrified-warmed porcine embryos.

	Embryo transfer procedure*					
	**S-30**		**NsDU-30**		**NsDU-40**	
	**Morulae**	**Blastocysts**	**Morulae**	**Blastocysts**	**Morulae**	**Blastocysts**
Recipients (N)	22	17	17	18	17	13
Parity (mean ± SD)	2.5 ± 1.5	2.1 ± 1.7	2.5 ± 1.9	2.4 ± 1.8	2.3 ± 1.5	2.2 ± 1.5
Pregnancy (25 d), N (%)	19 (86.4)^a^	15 (88.2)^a^	7 (41.2)^b^	9 (50.0)^b^	12 (70.6)^ab^	12 (92.3)^a^
Pregnancy (35 d), N (%)	16 (72.7)^a^	13 (76.5)^a^	5 (29.4)^b^	9 (50.0)^ab^	11 (64.7)^ab^	11 (84.6)^a^
Farrowing, N (%)	16 (72.7)^a^	13 (76.5)^a^	5 (29.4)^b^	9 (50.0)^ab^	11 (64.7)^ab^	10 (76.9)^a^
Total born (mean ± SD)	9.5 ± 2.8^a^	9.5 ± 2.5^a^	5.6 ± 2.2^b^	5.8 ± 2.7^b^	9.9 ± 1.4^a^	10.2 ± 2.2^a^
born alive (mean ± SD)	9.0 ± 2.5^a^	9.2 ± 2.5^a^	5.4 ± 2.3^b^	5.5 ± 2.6^b^	9.4 ± 1.3^a^	9.7 ± 2.3^a^
Piglet production efficiency# (%)	21.8^a^	23.4^a^	5.3^b^	9.1^c^	15.2^d^	16.0^ad^

^*^S-30: Surgical transfers with 30 vitrified-warmed embryos; NsDU-30: Non-surgical transfers with 30 vitrified-warmed embryos; NsDU-40: Non-surgical transfers with 40 vitrified-warmed embryos.

^#^Piglet production efficiency was calculated as the ratio of the number of live-born piglets to the number of embryos transferred to all recipients.

^a,b,c,d^Different letters in the same row indicate differences (P < 0.05).
